# The Lack of Self-Consciousness in Right Brain-Damaged Patients Can Be due to a Disconnection From the Left Interpreter: The DiLeI Theory

**DOI:** 10.3389/fpsyg.2019.00349

**Published:** 2019-02-28

**Authors:** Roberta Daini

**Affiliations:** Psychology, Università degli Studi di Milano Bicocca, Milan, Italy

**Keywords:** interpreter, anosognosia, corpus callosum, unilateral spatial neglect, self consciousness

## Introduction

Consciousness is a very complex topic and nonetheless one of the most attractive for philosophers, psychologists, and cognitive neuroscientists.

In general terms, the consciousness of the self can be described as the ability to reflect on oneself, one's own mental abilities, defined as the set of one's own sensations, perceptions, and thoughts.

Hereby this function and its deficits from a neuropsychological perspective will be dealt with. Many different theories and models on consciousness exist (e.g., Crick and Koch, 1998; Tononi and Edelman, 1998; Dehaene and Naccache, 2001) and what is lacking is the effort to put together different phenomena and apparently conflicting interpretations to provide a plausible account. Self-awareness is unitary, despite the multiple processes that underlie it, and this unity is fundamental to the formulation of goals, to plan and perform actions. An alteration of self-consciousness can, therefore, be seen as a loss of unity in self-perception and attaining a loss of effectiveness in formulating and achieving goals. A well-known and most well-studied example of self-consciousness deficit is anosognosia, the lack of consciousness about one's own sensory, motor or cognitive disabilities after a brain injury (e.g., Prigatano, 1996; Pia et al., 2004).

## The Paradox

Anosognosia is a symptom more associated with a right hemispheric lesion than a left one (e.g., Bisiach et al., 1986; Vossel et al., 2013; Pia et al., 2014). Moreover, right hemisphere damages consistently produce neuropathologies of the self, which are those related to the identity, the ego boundaries, and the relationship between the self and the external environment (e.g., Feinberg, 2011). Examples are the Capgras syndrome, delusional anosognosia, and somatoparaphrenia. This evidence could suggest a main role of the right hemisphere in the consciousness of the self-functioning. Nonetheless, studies on healthy participants tell a different story. When healthy participants are involved in tasks that require self-consciousness, as self-related judgments (Denny et al., 2012), functional neuroimaging studies showed a left hemisphere dominance.

Morin (2017) defined the inconsistency of results between healthy participants and right brain-damaged patients with anosognosia as the “self-awareness-anosognosia” paradox. He accounted for it by suggesting that the two types of studies measure aspects related to different processes, most likely associated with activity in distinct anatomical networks.

Moreover, not all brain-damaged patients' symptoms suggest a dominance of the right hemisphere for consciousness: for instance, the case of split-brain patients.

## The Interpreter

Fifty years of studies on split-brain patients (i.e., patients who underwent the resection of the corpus callosum) allowed Gazzaniga and his collaborators to frame the role of the left hemisphere in the consciousness of the self and to suggest the idea of an “Interpreter” (Volz and Gazzaniga, 2017). The typical observation is as follows: Visual stimuli tachistoscopically presented to the left hemifield are processed by the right hemisphere. Surprisingly, in this condition, split-brain patients verbally report that they do not see any stimulus. Even more surprisingly, when requested to point to a semantically related stimulus these patients point at the correct item with the left hand but do not verbally formulate the correct relationship. In healthy participants, information is transferred from the right hemisphere to the left one, interpreted and labeled. Results suggested that the “Interpreter” is located in the left hemisphere and it is strictly dependent not only on language but also on inferential reasoning (Volz and Gazzaniga, 2017).

The Interpreter represents a crucial aspect of consciousness and its localization, including left ventro-prefrontal cortex, left anterior and mid-insula, and dorsal caudate, is congruent with many studies about self-consciousness in healthy participants (e.g., Denny et al., 2012).

## The Underestimation of the Corpus Callosum Role

The studies on split-brain patients suggest an essential role not only of the left hemisphere but also of the corpus callosum in the conscious experience.

The corpus callosum is the largest fiber bundle of the human brain and connects the two cerebral hemispheres. It allows transfer of inputs from one hemisphere to the other and is involved in several sensory, motor, and cognitive functions.

Two main mechanisms have been described in consciousness: synchronization (Engel and Singer, 2001) and integration (e.g., Tononi, 2004). The corpus callosum is strictly involved in both processes.

Steinmann et al. (2018) found that inter-hemispheric functional connectivity was significantly enhanced during left ear/right hemisphere conscious processing of auditory stimuli as compared to right ear/left hemisphere conscious processing of auditory stimuli. They found that conscious reports require causal interhemispheric inputs from the right to the left auditory cortices and that this interaction is mediated by synchronized gamma-band oscillations.

Studies on split-brain patients (Volz and Gazzaniga, 2017) and healthy participants (e.g., Banich and Belger, 1990) suggest that the corpus callosum cannot be thought of as a simple and passive information transfer channel. It is true, instead, that it is a complex set of fibers with different components acting separately and that the degree to which the cerebral hemispheres elaborate information independently or jointly is a relative phenomenon rather than absolute (Bloom and Hynd, 2005).

Moreover, the corpus callosum is thought to be a “symmetrical” connection between the left and right hemisphere. DTI studies on healthy participants found numerous asymmetries in the callosal connections; at the splenial level the direction of the connection, for the most part, is from the right to the left hemisphere rather than the opposite direction (Putnam et al., 2010; Iwabuchi et al., 2011).

## A new Proposal

If the interpreter is fundamental for the consciousness of the self-functioning and it is lateralized in the left hemisphere, why is anosognosia more often associated with lesions of the right hemisphere?

A possible interpretation is that the left hemisphere is relevant for self-consciousness and that a damage to the right hemisphere does not affect the areas strictly involved in self-consciousness but instead induces a “disconnection” between what is processed in the right hemisphere and the self-consciousness-related system in the left hemisphere. This hypothesis allows to put together many “distinct” phenomena and theories; first of all, the Gazzaniga's “interpreter,” and calls for the double role of the corpus callosum.

On the one hand, the corpus callosum is one of most crucial structures for the synchronization and integration of sensory, motor and cognitive processes (e.g., Paul et al., 2007); mechanisms that are considered the neural basis of conscious perception.

On the other hand, callosal fibers have the task not only of transferring information from one hemisphere to the other one but also of inhibiting contralateral representations in “competitive” contexts (e.g., Bloom and Hynd, 2005). The more the function is lateralized, the more the connection is inhibitory to ensure that the dominant hemisphere is activated (Cook, 1984). Moreover, the fibers of the corpus callosum are not symmetrical. A lesion of the right hemisphere involving the white matter could result in a desynchronization/inhibition by the interhemispheric fibers of the left hemisphere, especially for strongly lateralized functions.

In this frame, a lesion of the left hemisphere can disrupt only intrahemispheric connections relevant for conscious content, while right hemispheric lesions affect both intrahemispheric and interhemispheric connectivity.

Although a damage to the corpus callosum fibers can be the reason for a disconnection deficit of anosognosia, a lesion within the right hemisphere (not necessarily involving the corpus callosum) determines an effect over the equilibrium of connectivity between the two hemispheres and this, in turn, alters the synchronization and integration between the processes that started within each hemisphere.

An essential role in anosognosia is attributed to the impairment of anatomo-functionally discrete monitoring systems (Berti et al., 2005; Vallar and Ronchi, 2006; Moro et al., 2011). The current proposal suggests that the self-monitoring is the consequence of the same processing responsible for conscious experience, going from the right hemisphere to the left Interpreter, which is impaired in those patients.

## The Case of Unilateral Spatial Neglect (NSU): a Bridge Between Kinsbourne and Heilman's Theories

Brain-damaged patients affected by unilateral spatial neglect (USN) fail to report, respond to, and orient to stimuli presented on the side of space contralateral to the lesion. USN can also be described as a deficit of consciousness of the contralesional space, either of the own body or the external environment. It is also characterized by anosognosia, i.e., patients are not aware of having USN, and it is more severe after right hemisphere damage (Weintraub and Mesulam, 1987).

Two of the leading theories proposed to account for unilateral spatial neglect (USN) are those of Kinsbourne (1970) and Heilman and Valenstein (1979), which start from opposite assumptions. The former states that the left hemisphere is dominant for visuo-spatial attention and a right damage induces a rightward bias because it emphasizes the imbalance; the latter suggests that the right hemisphere is dominant for visuo-spatial attention and is entailed with the entire space, whereas the left hemisphere is involved only in orienting attention to the contralateral hemispace. Some data support Kinsbourne's model (e.g., Corbetta et al., 2005; Salatino et al., 2014), while others support the Heilman's one (e.g., Ricci et al., 2012; Bagattini et al., 2015).

According to the Disconnection from the Left Interpreter (DiLeI) theory, the dominance of the right hemisphere for visuo-spatial attention is compatible with the Kinsbourne's explanation of USN in terms of an attentional vector of the left hemisphere toward the ipsilesional side of space.

The right lesion, indeed, could affect the right-toward-left-hemisphere directional asymmetry of callosal fibers by reducing the inhibitory effect over the left–toward-right activity. The first consequence could be a reduction in interhemispheric functional connectivity of dorsal attention and sensory-motor networks shown by Baldassarre et al. (2014) for right brain-damaged patients with resting state fMRI. The authors measured spontaneous brain activity in a resting state functional connectivity mapping study and found a reduced interhemispheric functional connectivity for the dorsal attention and sensory-motor networks. This pattern was stronger in patients with right- hemisphere as compared to left-hemisphere damage, with neglect more than without neglect and, finally, correlated with the NSU tests performance.

Accordingly, disruption of callosal connections causes more severe neglect (Bozzali et al., 2012), and severely reduces interhemispheric functional connectivity (Johnston et al., 2008).

The DiLeI theory proposes that the “isolation” of the right hemisphere: (1) impedes the perceptual processing from reaching the Interpreter in the left hemisphere and then induces the lack of consciousness for the stimuli presented in the left hemispace; (2) enhances the activity of the left hemisphere and then the saliency of the stimuli in the right side of space and their power of orienting attention.

I am not supporting the idea that USN is due to the disconnection of the right hemisphere with the Interpreter. There are other specific mechanisms underlying USN that are linked with the right hemisphere functioning. Nevertheless, this disconnection can explain some phenomena linked with USN (i.e., the anosognosia for neglect, the conscious perception of only the stimuli processed by the left hemisphere and the perceptual saliency of the ipsilesional stimuli).

## Conclusions

The DiLeI theory states that the lack of access to the Interpreter's system, and therefore, the lack of integration with the other contents of the conscience would explain the greater incidence of deficits for right brain damages for functions not necessarily lateralized on the right. In other words, the consciousness would not depend (only) on modules located in the right hemisphere, nor in the left, but on the integration of the two hemispheres through the corpus callosum.

This theory has the advantage of being applicable to many areas, from psychiatry to personality and cognitive psychology in general. Schizophrenia, for example, is considered to be the consequence of multiple dysfunctional mechanisms, including the one that underlies information binding (Tononi and Edelman, 2000) and self-monitoring (Frith, 1992). In this context, the mechanisms of information binding and self-monitoring would depend on the interpreter circuit and form the connection between the two hemispheres. Neuroimaging techniques, indeed, have shown both the functional alteration of the cortico-subcortical circuits of the left hemisphere (fronto-temporal areas, insula, cerebellum, thalamus), neural correlate of the Interpreter, and a hypoactivation of the left hemisphere, and hyperactivation of the right (e.g., Trimble and George, 2010). Nonetheless, this interpretation is suited to the disconnection syndrome theory by Friston and Frith (1995).

The DiLeI theory needs, of course, to be validated, and I predict that it will have heuristic meaning to “interpret” multiple phenomena with discrete mechanisms and processes.

## Author Contributions

The author confirms being the sole contributor of this work and has approved it for publication.

### Conflict of Interest Statement

The author declares that the research was conducted in the absence of any commercial or financial relationships that could be construed as a potential conflict of interest.
